# Supplementary material for: Description of Gopheromyces tardescens, gen. nov., sp. nov., Gigasporangiomyces pilosus, gen. nov., sp. nov., Kelyphomyces adhaerens, gen. nov., sp. nov., and proposal of Testudinimycetaceae, fam. nov
Source: Int J Syst Evol Microbiol. 2026 Jun 19;76(6):007196. doi: 10.1099/ijsem.0.007196 (PMC13286287; doi:10.1099/ijsem.0.007196)
Supplement: Supplementary Material 1. [file ijsem-76-07196-s001.pdf]

Description of *Gopheromyces tardescens*, gen. nov., sp.nov., *Gigasporangiomyces pilosus,* gen. nov., sp.nov., *Kelyphomyces adhaerens,* gen. nov., sp. nov., and proposal of *Testudinimycetaceae* fam. nov.

Alexandria Morris[1], Taylor Mills[1], Samuel L. Miller[1], Carrie J. Pratt[1,2], Stephen Marek[3], Yan Wang[2], Mostafa S. Elshahed[1], Noha H. Youssef[1], Julia Vinzelj[1*]

[1]Department of Microbiology and Molecular Genetics, Oklahoma State University, Stillwater, OK, USA

[2]Department of Biological Sciences, University of Toronto Scarborough, Toronto, ON, Canada

[3]Department of Entomology and Plant Pathology, Oklahoma State University, Stillwater, OK, USA

*Corresponding Author: Address: 1110 S. Innovation Way, Stillwater, OK, USA. Phone: +1 (405) 744-3005. Email: julia.vinzelj@okstate.edu

Supplementary

BioProject number: PRJNA1345044 **GenBank accession numbers:** OQ382944-OQ382956, PX576123-PX576134

Keywords: *Neocallimastigomycota*; anaerobic fungi; tortoises; AAI; molecular dating analysis

Table S1. Strains of *Neocallimastigomycota* obtained in this study. RFC = rumen fluid cellobiose medium [19] + SG = supplementation of 0.1% w/v switchgrass. C = cloning, G = genomics, T = transcriptomics, TE = temperature experiments, SE = substrate experiments.

Table S2. Results of D2 LSU-based culture-independent diversity surveys on the two sulcata tortoises used for isolation of novel strains SR0.6 and TM0.3.

Table S3. Measurements of microscopic features for the three novel *Neocallimastigomycota* strains obtained in this study. For measurements of sporangia, "length" refers to the straight line between the base of the sporangium (where the sporangiophore sits) to the opposite edge, and "width" refers to the widest edge-to-edge measurement perpendicular to that line. Zoospores are measured similarly (one line from the base where the flagellum attaches to the opposite edge, and one line perpendicular to that from edge to edge at the widest point). For the elongated stalks of GXA2, "length" refers to the measurement from the base to the tip, and width refers to perpendicular measurements from edge to edge (multiple per stalk). For filaments/hyphae measurements, no length measurements were taken, only measurements of the width.

Table S4. Substrate and temperature preferences for the three novel *Neocallimastigomycota* strains obtained in this study. Redness and turbidity of the medium as well as biomass production of the fungi was rated on a scale from 0 (none) to 4 (a lot).

Figure S1. Growth curves for the three novel *Neocallimastigomycota* isolates (GXA2, SR0.6, TM0.3) grown in rumen fluid media containing either cellobiose (SR0.6, TM0.3) or lactose (GXA2) as substrate at 35 °C. Solid lines represent the average cumulative gas pressure (PSI) and dashed lines the average of the visual growth evaluation (from 0, no growth, to 4, very good growth). Measurements were taken from the same four tubes per strain at the indicated time points after subculture.

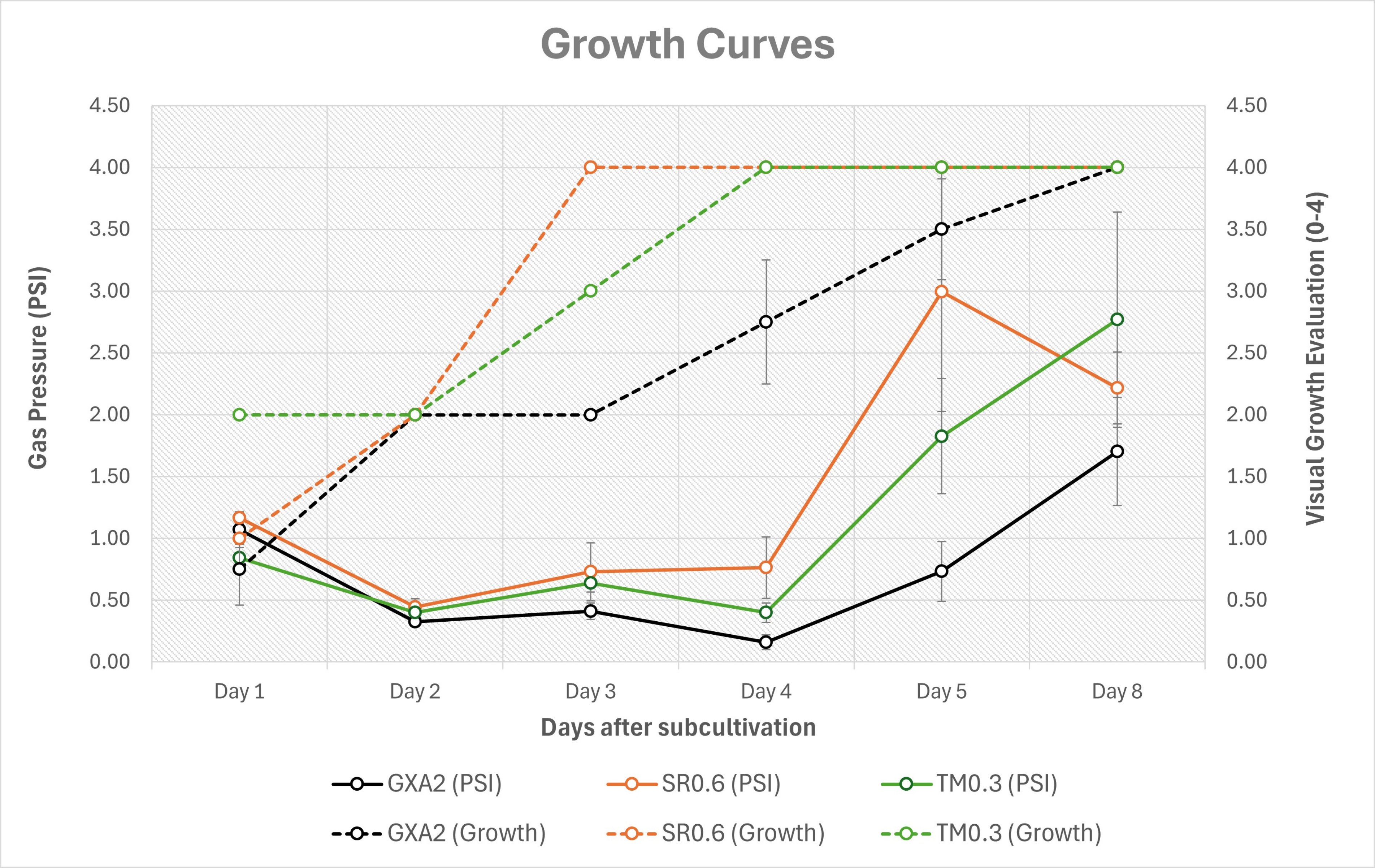
Growth Curves
Gas Pressure (PSI)
Visual Growth Evaluation (0-4)
0.00
0.50
1.00
1.50
2.00
2.50
3.00
3.50
4.00
4.50
Day 1
Day 2
Day 3
Day 4
Day 5
Day 8
Days after subcultivation
GXA2 (PSI)
SR0.6 (PSI)
TM0.3 (PSI)
GXA2 (Growth)
SR0.6 (Growth)
TM0.3 (Growth)

Figure S2. Growth of the three novel *Neocallimastigomycota* isolates (GXA2, SR0.6, TM0.3) in rumen fluid media containing various substrates. Bars represent the average gas pressure (PSI), shorter horizontal lines represent the average of the visual growth evaluation (from 0, no growth, to 4, very good growth), and the black horizontal lines represent the average gas pressure of the negative controls (uninoculated tubes). **(A)** Results after the first transfer on the various substrates. **(B)** Average after the last transfer on the various substrates. **(C)** Average over the whole experiment.

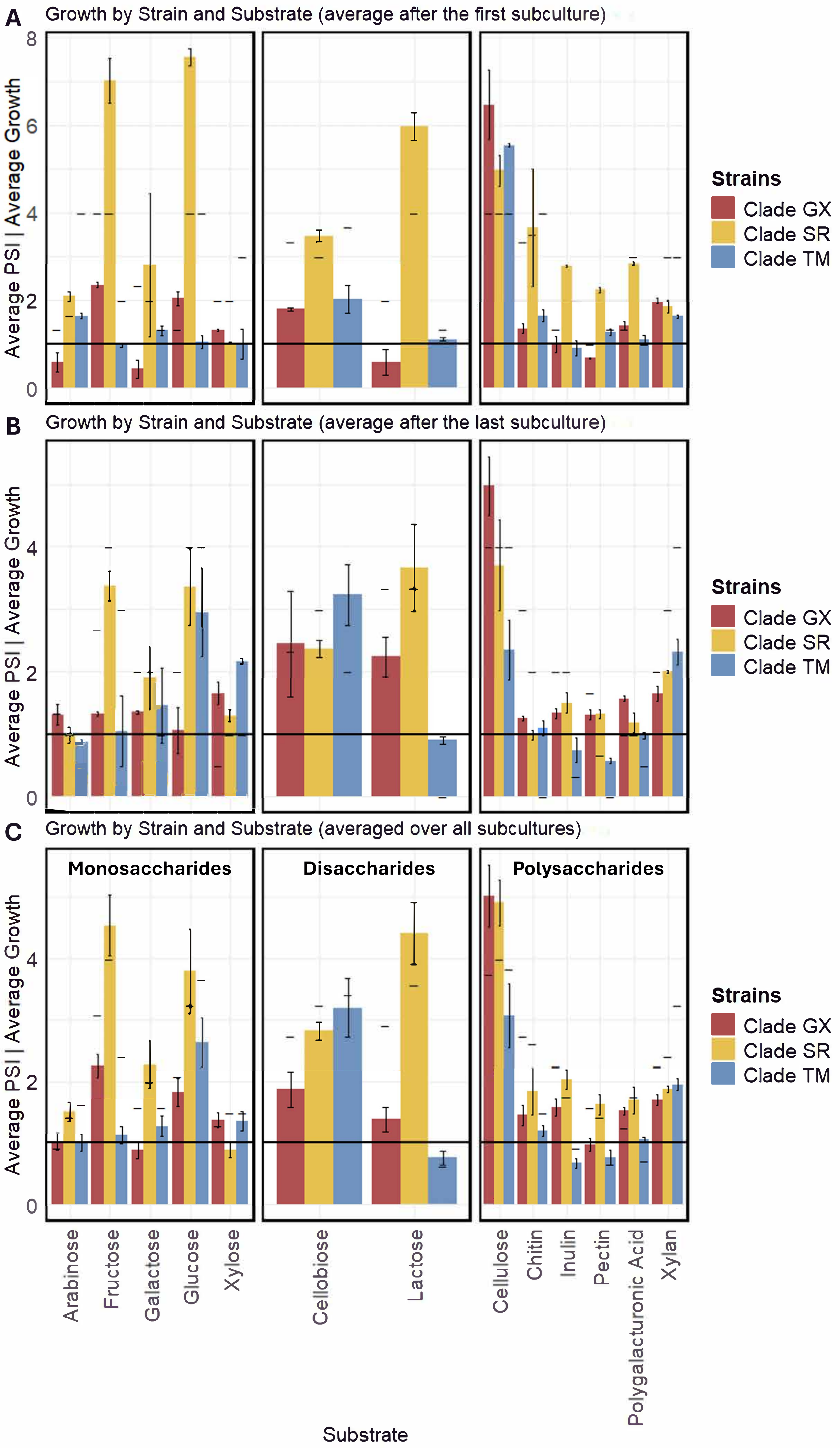

A
Growth by Strain and Substrate (average after the first subculture)
B
Growth by Strain and Substrate (average after the last subculture)
C
Growth by Strain and Substrate (averaged over all subcultures)
Monosaccharides
Disaccharides
Polysaccharides
Average PSI | Average Growth
Strains
Clade GX
Clade SR
Clade TM
Arabinose
Fructose
Galactose
Glucose
Xylose
Cellobiose
Lactose
Cellulose
Chitin
Inulin
Pectin
Polygalacturonic Acid
Xylan
Substrate

Figure S3. Growth of the three novel *Neocallimastigomycota* isolates (GXA2, SR0.6, TM0.3) at various temperatures. Strains were either grown in rumen fluid medium containing cellobiose (SR0.6, TM0.3) or lactose (GXA2) at different temperatures. Bars represent the average gas pressure (PSI), shorter horizontal lines represent the average of the visual growth evaluation (from 0, no growth, to 4, very good growth), and the black horizontal lines represent the average gas pressure of the negative controls (uninoculated tubes). **(A)** Results after the first transfer at the various temperatures. **(B)** Average after the last transfer at the various temperatures. For strain GXA2 on 22 and 39 °C the data after the second subculture is displayed. **(C)** Average over the whole experiment. For strain GXA2 on 22 and 39 °C the data was averaged just over the first and second subculture since it was dead and not further subcultured.

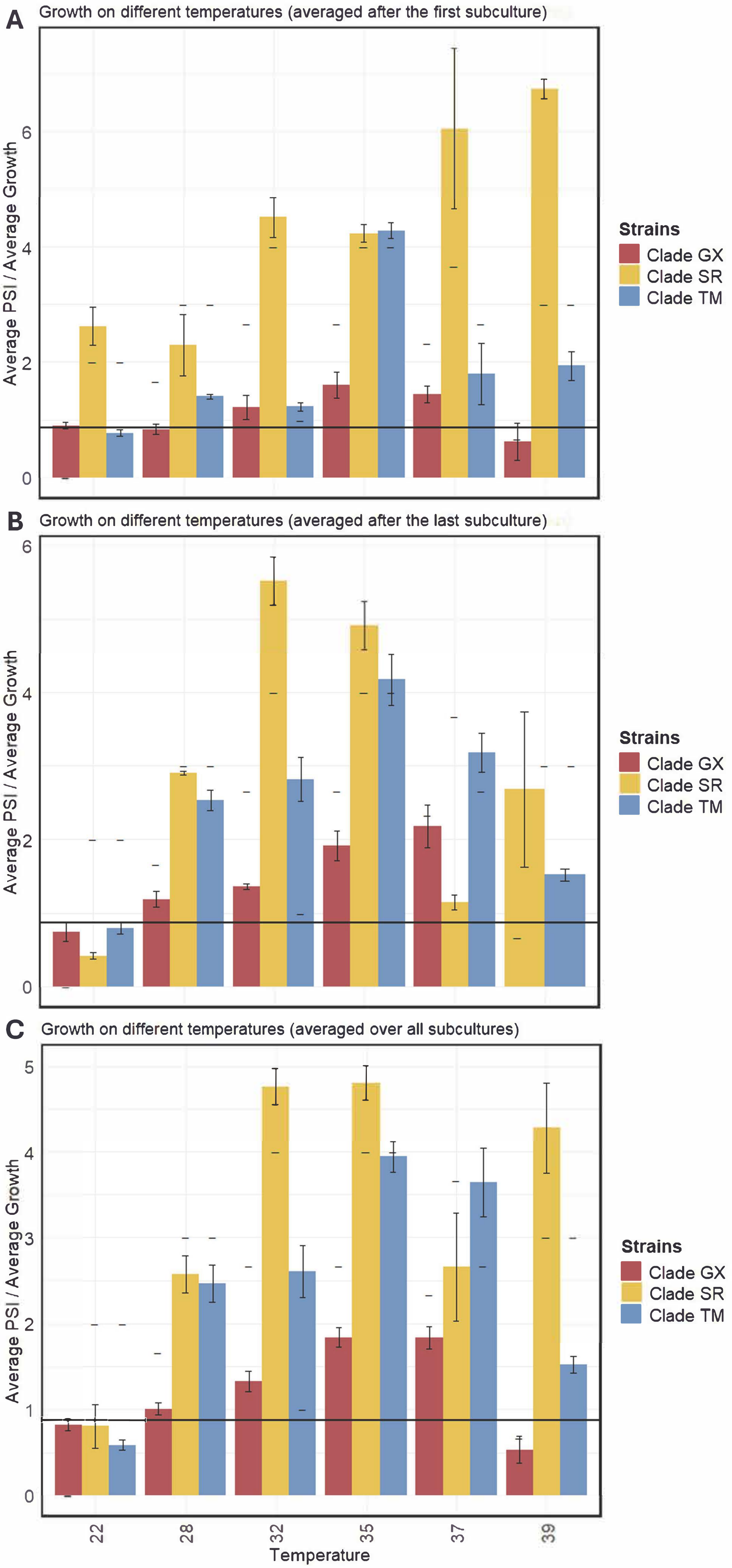
A
Growth on different temperatures (averaged after the first subculture)
B
Growth on different temperatures (averaged after the last subculture)
C
Growth on different temperatures (averaged over all subcultures)
Average PSI / Average Growth
Temperature
22
28
32
35
37
39
Strains
Clade GX
Clade SR
Clade TM
